# {μ-1,2-Bis[bis(4-meth­oxy­phen­yl)phosphan­yl]-1,2-dimethyl­hydrazine-κ^2^ *P*:*P*′}bis­[chloridogold(I)] tetra­hydro­furan disolvate

**DOI:** 10.1107/S1600536811028856

**Published:** 2011-07-30

**Authors:** Frederik H. Kriel, Manuel A. Fernandes, Judy Coates

**Affiliations:** a AuTEK, Mintek, Private Bag X3015, Randburg, 2125, South Africa; b Molecular Science Institute, School of Chemistry, University of the Witwatersrand, PO Wits, 2050, Johannesburg, South Africa

## Abstract

The title compound, [Au_2_Cl_2_(C_30_H_34_N_2_O_4_P_2_)]·2C_4_H_8_O, is formed from a bidentate phosphine ligand complexed to two almost linearly coordinated gold(I) atoms [P—Au—Cl = 175.68 (3) Å]. The nuclei are 3.122 (2) Å apart. The mol­ecule exhibits a twofold rotation axis.

## Related literature

For the synthesis of the parent ligand and related structures utilizing alternative metals, see: Reddy *et al.* (1994 ▶, 1995 ▶); Slawin *et al.* (2002 ▶); Kriel *et al.* (2010*a* ▶,*b* ▶, 2011*a* ▶,*b* ▶). For Au⋯Au inter­actions, see: Holleman & Wiberg (2001 ▶).
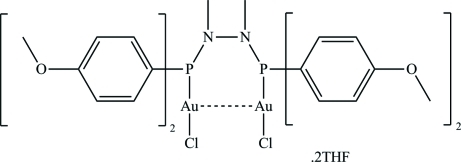


## Experimental

### 

#### Crystal data

[Au_2_Cl_2_(C_30_H_34_N_2_O_4_P_2_)]·2C_4_H_8_O
*M* *_r_* = 1157.57
Monoclinic, 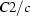

*a* = 23.208 (5) Å
*b* = 9.080 (5) Å
*c* = 20.220 (5) Å
β = 92.414 (5)°
*V* = 4257 (3) Å^3^
*Z* = 4
Mo *K*α radiation
μ = 7.13 mm^−1^
*T* = 173 K
0.58 × 0.45 × 0.10 mm

#### Data collection

Bruker SMART CCD area-detector diffractometer
Absorption correction: multi-scan (*SADABS*; Bruker, 1999 ▶) *T*_min_ = 0.044, *T*_max_ = 0.567
33198 measured reflections
5264 independent reflections
4679 reflections with *I* > 2σ(*I*)
*R*_int_ = 0.040

#### Refinement

*R*[*F* ^2^ > 2σ(*F* ^2^)] = 0.019
*wR*(*F* ^2^) = 0.049
*S* = 1.04
5264 reflections
235 parameters
H-atom parameters constrained
Δρ_max_ = 1.08 e Å^−3^
Δρ_min_ = −0.80 e Å^−3^

### 

Data collection: *SMART-NT* (Bruker, 1998 ▶); cell refinement: *SAINT-Plus* (Bruker, 1999 ▶); data reduction: *SAINT-Plus*; program(s) used to solve structure: *SHELXS97* (Sheldrick, 2008 ▶); program(s) used to refine structure: *SHELXL97* (Sheldrick, 2008 ▶); molecular graphics: *ORTEP-3* (Farrugia, 1997 ▶) and *Mercury* (Macrae *et al.*, 2008 ▶); software used to prepare material for publication: *WinGX* (Farrugia, 1999 ▶).

## Supplementary Material

Crystal structure: contains datablock(s) I, global. DOI: 10.1107/S1600536811028856/bg2412sup1.cif

Structure factors: contains datablock(s) I. DOI: 10.1107/S1600536811028856/bg2412Isup2.hkl

Additional supplementary materials:  crystallographic information; 3D view; checkCIF report

## Figures and Tables

**Fig. 1.**
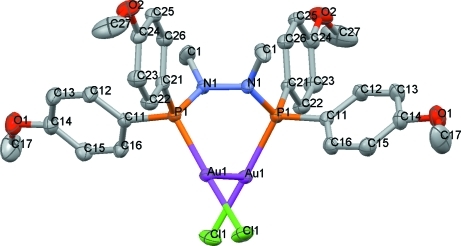
: Molecular structure of I drawn with displacement ellipsoids at the 50% probability level. Hydrogen atoms and solvent THF have been omitted for clarity.

**Fig. 2.**
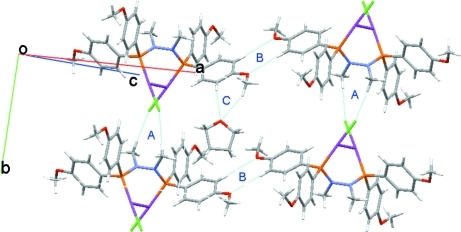
: Packing of compound I showing short contacts.

## supplementary crystallographic information

### Comment

The title compound (C_30_H_34_Au_2_Cl_2_N_2_O_4_P_2_.2(C_4_H_8_O)), formed from a bidentate phosphine ligand complexed to two linear gold(I) nuclei, readilly crystalizes out of dichloromethane (DCM) with the addition of a few drops of tetrahydrofuran (THF). The crystal structure includes a THF solvent molecule. The complex molecule is bisected by a two fold axis throul the N-N' and Au-Au' lines (Fig 1). Gold(I) has an almost linear coordination with a P—Au—Cl angle of 175.68 (3) °. The Au—Au distance within the complex is 3.122 (2) Å, well within the range of aurophilic interactions (described in Holleman *et al.*, 2001, as being normally between 2.7 Å and 3.4 Å). Other bond lengths are within expected ranges.

The structure exhibits columns of complexes arranged head-to-tail along b, forming channels filled with THF. There is an intercolumnar contact involving chloride atoms in one molecule and hydrogen atoms on the methyl substituted hydrazine bridge of a neighbouring one, in the same column (Cl1···H1c^i^: 2.892Å, (i): 1-x,1+y,1/2-z, site A in Fig 2). There are also weak intercolumnar H-bonding contacts (O1···H13^ii^: 2.629Å, (ii): 3/2-x,-1/2-y,1-z), site B in Fig 2). Finally, the THF solvato molecule is weakly attached to the columns by a pair of O···H contacts (O3···H15:2.608 Å; O3···H15b: 2.557 Å) (site C in Fig. 2).

### Experimental

The complex was synthesized by dissolving tetrahydrothiophenegold(I) chloride [(THT)AuCl] in DCM and adding 0.5 equivalents of the corresponding ligand (bis(di(4-methoxyphenyl)phosphino)-1,2-dimethylhydrazine. The addition of a few drops of THF led to the growth of crystals suitable for use in single-crystal X-Ray analysis. The presence of THF during the initial complexation led to undesirable side products as a result of the breakdown of the ligand.

### Refinement

The H atoms were positioned geometrically and allowed to ride on their respective parent atoms, with C—H = 0.93 (CH) or 0.96 (CH_3_) Å, and with *U*_eq_ = 1.2 (CH) or 1.5 (CH_3_) *U*_eq_(C).

### Crystal data

**Table:**

[Au_2_Cl_2_(C_30_H_34_N_2_O_4_P_2_)]·2C_4_H_8_O	*F*(000) = 2248
*M**_r_* = 1157.57	*D*_x_ = 1.806 Mg m^−^^3^
Monoclinic, *C*2/*c*	Mo *K*α radiation, λ = 0.71073 Å
Hall symbol: -C 2yc	Cell parameters from 7327 reflections
*a* = 23.208 (5) Å	θ = 2.4–28.3°
*b* = 9.080 (5) Å	µ = 7.13 mm^−^^1^
*c* = 20.220 (5) Å	*T* = 173 K
β = 92.414 (5)°	Plate, colourless
*V* = 4257 (3) Å^3^	0.58 × 0.45 × 0.10 mm
*Z* = 4	

### Data collection

**Table:**

Bruker SMART CCD area-detector diffractometer	5264 independent reflections
Radiation source: fine-focus sealed tube	4679 reflections with *I* > 2σ(*I*)
graphite	*R*_int_ = 0.040
phi and ω scans	θ_max_ = 28.3°, θ_min_ = 1.8°
Absorption correction: multi-scan (*SADABS*; Bruker, 1999)	*h* = −30→30
*T*_min_ = 0.044, *T*_max_ = 0.567	*k* = −12→11
33198 measured reflections	*l* = −25→26

### Refinement

**Table:**

Refinement on *F*^2^	Primary atom site location: structure-invariant direct methods
Least-squares matrix: full	Secondary atom site location: difference Fourier map
*R*[*F*^2^ > 2σ(*F*^2^)] = 0.019	Hydrogen site location: inferred from neighbouring sites
*wR*(*F*^2^) = 0.049	H-atom parameters constrained
*S* = 1.04	*w* = 1/[σ^2^(*F*_o_^2^) + (0.0199*P*)^2^ + 7.2119*P*] where *P* = (*F*_o_^2^ + 2*F*_c_^2^)/3
5264 reflections	(Δ/σ)_max_ = 0.004
235 parameters	Δρ_max_ = 1.08 e Å^−^^3^
0 restraints	Δρ_min_ = −0.80 e Å^−^^3^

### Special details

**Table:**

Experimental. Reaction: bis(di(4-methoxyphenyl)phosphino)-1,2-dimethylhydrazine: 167 mg (0.29 mmol), (THT)AuCl: 200 mg (0.57 mmol), dichloromethane: 5 ml, tetrahydrofuran: few drops, Yield: 75%. Colourless to grey crystals. ^1^H NMR: (CDCl_3_, 300 MHz) δ_H_ 7.79 (t, Arom, *J* = 8.2 Hz, 4H) 7.41 (t, Arom, *J* = 8.2 Hz, 4H), 6.97 (d, Arom, *J* = 7.6 Hz, 4H), 6.81 (d, Arom, *J* = 7.6 Hz, 4H), 3.86 (s, OMe, 6H), 3.81 (s, OMe, 6H), 2.69 (d, NCH_3_, ^3^*J* (^1^H-^31^P) *=* 5.8 Hz, 6H). ^13^C NMR: (CDCl_3_, 75 MHz) δ_C_ 163.5 (d, Arom, *J =* 33.4 Hz), 135.9 (m, Arom), 115.3 (m, Arom), 55.52 and 55.45 (s, OCH_3_), 35.1 (s, NCH_3_). ^31^P NMR:(CDCl_3_, 121 MHz) δ_P_ 85.1. MS: 977 (83%, *M* - Cl), 245 (80%, P(PhOMe)_2_). EA:Calc: (Au_2_Cl_2_P_2_O_4_N_2_C_30_H_34_) C 35.56%, H 3.38%, N 2.76%. Found: C 36.78%, H 3.79%, N 2.47%. MP: 166 – 170 °C.
Geometry. All e.s.d.'s (except the e.s.d. in the dihedral angle between two l.s. planes) are estimated using the full covariance matrix. The cell e.s.d.'s are taken into account individually in the estimation of e.s.d.'s in distances, angles and torsion angles; correlations between e.s.d.'s in cell parameters are only used when they are defined by crystal symmetry. An approximate (isotropic) treatment of cell e.s.d.'s is used for estimating e.s.d.'s involving l.s. planes.
Refinement. Refinement of *F*^2^ against ALL reflections. The weighted *R*-factor *wR* and goodness of fit *S* are based on *F*^2^, conventional *R*-factors *R* are based on *F*, with *F* set to zero for negative *F*^2^. The threshold expression of *F*^2^ > σ(*F*^2^) is used only for calculating *R*-factors(gt) *etc*. and is not relevant to the choice of reflections for refinement. *R*-factors based on *F*^2^ are statistically about twice as large as those based on *F*, and *R*- factors based on ALL data will be even larger.

### Fractional atomic coordinates and isotropic or equivalent isotropic displacement parameters (Å^2^)

**Table:**

	*x*	*y*	*z*	*U*_iso_*/*U*_eq_	
C1	0.56719 (14)	−0.3148 (4)	0.26405 (15)	0.0437 (8)	
H1A	0.5974	−0.3050	0.2976	0.066*	
H1B	0.5830	−0.3040	0.2212	0.066*	
H1C	0.5497	−0.4102	0.2673	0.066*	
C11	0.58836 (10)	−0.0866 (3)	0.37553 (12)	0.0238 (5)	
C12	0.60878 (11)	−0.1775 (3)	0.42700 (13)	0.0316 (6)	
H12	0.5836	−0.2413	0.4473	0.038*	
C13	0.66594 (12)	−0.1732 (3)	0.44792 (14)	0.0383 (6)	
H13	0.6794	−0.2351	0.4818	0.046*	
C14	0.70353 (11)	−0.0767 (3)	0.41852 (15)	0.0360 (6)	
C15	0.68388 (11)	0.0165 (3)	0.36871 (13)	0.0337 (6)	
H15	0.7089	0.0826	0.3496	0.040*	
C16	0.62614 (11)	0.0104 (3)	0.34742 (12)	0.0287 (5)	
H16	0.6127	0.0727	0.3137	0.034*	
C17	0.80122 (14)	−0.0045 (5)	0.4075 (2)	0.0714 (12)	
H17A	0.8385	−0.0180	0.4291	0.107*	
H17B	0.7916	0.0983	0.4069	0.107*	
H17C	0.8019	−0.0405	0.3629	0.107*	
C21	0.47826 (10)	−0.2085 (3)	0.40186 (12)	0.0237 (5)	
C22	0.45316 (11)	−0.1382 (3)	0.45396 (13)	0.0287 (5)	
H22	0.4534	−0.0358	0.4559	0.034*	
C23	0.42742 (12)	−0.2182 (3)	0.50370 (13)	0.0336 (6)	
H23	0.4113	−0.1699	0.5390	0.040*	
C24	0.42619 (11)	−0.3700 (3)	0.49983 (14)	0.0299 (6)	
C25	0.45039 (11)	−0.4422 (3)	0.44695 (13)	0.0283 (5)	
H25	0.4490	−0.5444	0.4442	0.034*	
C26	0.47638 (11)	−0.3615 (3)	0.39880 (13)	0.0271 (5)	
H26	0.4929	−0.4100	0.3638	0.032*	
C27	0.3811 (2)	−0.3925 (4)	0.6031 (2)	0.0793 (16)	
H27A	0.3653	−0.4664	0.6310	0.119*	
H27B	0.3517	−0.3223	0.5906	0.119*	
H27C	0.4122	−0.3431	0.6267	0.119*	
C31	0.7520 (3)	0.4212 (6)	0.2921 (3)	0.1022 (19)	
H31A	0.7121	0.4137	0.2760	0.123*	
H31B	0.7767	0.3897	0.2574	0.123*	
C32	0.7659 (4)	0.5757 (6)	0.3128 (3)	0.127 (3)	
H32A	0.7899	0.6231	0.2809	0.152*	
H32B	0.7309	0.6330	0.3167	0.152*	
C33	0.7965 (3)	0.5625 (8)	0.3765 (3)	0.124 (2)	
H33A	0.8348	0.6048	0.3751	0.149*	
H33B	0.7757	0.6114	0.4108	0.149*	
C34	0.7993 (5)	0.4148 (8)	0.3874 (6)	0.240 (8)	
H34A	0.7915	0.3953	0.4333	0.288*	
H34B	0.8381	0.3809	0.3799	0.288*	
N1	0.52377 (8)	−0.2011 (2)	0.27315 (10)	0.0240 (4)	
O1	0.75927 (9)	−0.0834 (3)	0.44229 (12)	0.0507 (6)	
O2	0.40217 (9)	−0.4604 (2)	0.54504 (10)	0.0417 (5)	
O3	0.76126 (16)	0.3366 (4)	0.3477 (2)	0.0940 (11)	
P1	0.51542 (3)	−0.10079 (7)	0.34180 (3)	0.02187 (13)	
Cl1	0.41744 (3)	0.31848 (8)	0.29583 (4)	0.04364 (17)	
Au1	0.470213 (4)	0.109672 (10)	0.317798 (4)	0.02473 (4)	

### Atomic displacement parameters (Å^2^)

**Table:**

	*U*^11^	*U*^22^	*U*^33^	*U*^12^	*U*^13^	*U*^23^
C1	0.0542 (17)	0.0412 (18)	0.0345 (15)	0.0252 (15)	−0.0130 (13)	−0.0115 (13)
C11	0.0244 (11)	0.0244 (13)	0.0223 (12)	0.0004 (9)	−0.0026 (9)	−0.0014 (9)
C12	0.0331 (13)	0.0307 (15)	0.0305 (13)	−0.0038 (11)	−0.0052 (10)	0.0078 (11)
C13	0.0369 (14)	0.0387 (17)	0.0382 (15)	−0.0021 (13)	−0.0091 (11)	0.0123 (13)
C14	0.0283 (12)	0.0405 (17)	0.0384 (16)	−0.0014 (12)	−0.0084 (11)	0.0013 (13)
C15	0.0300 (13)	0.0370 (16)	0.0342 (14)	−0.0065 (11)	0.0013 (11)	0.0036 (12)
C16	0.0310 (12)	0.0309 (14)	0.0241 (12)	0.0002 (11)	−0.0003 (10)	0.0053 (10)
C17	0.0293 (15)	0.090 (3)	0.094 (3)	−0.0153 (18)	−0.0118 (17)	0.027 (2)
C21	0.0246 (11)	0.0223 (13)	0.0239 (12)	−0.0024 (9)	−0.0029 (9)	0.0021 (9)
C22	0.0330 (13)	0.0221 (13)	0.0309 (14)	−0.0019 (10)	0.0028 (10)	−0.0007 (10)
C23	0.0398 (14)	0.0312 (15)	0.0304 (14)	0.0001 (12)	0.0081 (11)	−0.0017 (11)
C24	0.0275 (12)	0.0299 (15)	0.0326 (14)	−0.0018 (10)	0.0026 (10)	0.0079 (11)
C25	0.0303 (12)	0.0209 (13)	0.0333 (14)	−0.0026 (10)	−0.0026 (10)	0.0009 (11)
C26	0.0287 (12)	0.0235 (13)	0.0289 (13)	0.0007 (10)	−0.0001 (10)	−0.0006 (10)
C27	0.114 (4)	0.055 (3)	0.074 (3)	0.018 (2)	0.062 (3)	0.026 (2)
C31	0.154 (6)	0.091 (4)	0.064 (3)	−0.039 (4)	0.022 (3)	0.004 (3)
C32	0.229 (8)	0.068 (4)	0.085 (4)	−0.009 (4)	0.042 (5)	0.008 (3)
C33	0.185 (7)	0.100 (5)	0.090 (5)	−0.055 (5)	0.018 (4)	−0.011 (4)
C34	0.248 (11)	0.096 (6)	0.356 (17)	−0.057 (6)	−0.225 (12)	0.061 (7)
N1	0.0299 (10)	0.0220 (11)	0.0196 (10)	0.0040 (8)	−0.0054 (8)	−0.0014 (8)
O1	0.0295 (10)	0.0591 (15)	0.0620 (15)	−0.0089 (10)	−0.0154 (10)	0.0151 (12)
O2	0.0475 (11)	0.0327 (12)	0.0462 (12)	0.0009 (9)	0.0172 (9)	0.0131 (9)
O3	0.100 (2)	0.061 (2)	0.118 (3)	−0.0244 (18)	−0.025 (2)	0.022 (2)
P1	0.0249 (3)	0.0202 (3)	0.0203 (3)	−0.0005 (2)	−0.0015 (2)	0.0000 (2)
Cl1	0.0563 (4)	0.0254 (4)	0.0495 (4)	0.0139 (3)	0.0055 (3)	0.0033 (3)
Au1	0.03095 (6)	0.01863 (6)	0.02458 (6)	0.00191 (4)	0.00082 (4)	−0.00061 (3)

### Geometric parameters (Å, °)

**Table:**

C1—N1	1.460 (3)	C24—O2	1.365 (3)
C1—H1A	0.9600	C24—C25	1.393 (4)
C1—H1B	0.9600	C25—C26	1.378 (4)
C1—H1C	0.9600	C25—H25	0.9300
C11—C16	1.382 (4)	C26—H26	0.9300
C11—C12	1.395 (4)	C27—O2	1.430 (4)
C11—P1	1.803 (2)	C27—H27A	0.9600
C12—C13	1.376 (4)	C27—H27B	0.9600
C12—H12	0.9300	C27—H27C	0.9600
C13—C14	1.388 (4)	C31—O3	1.371 (6)
C13—H13	0.9300	C31—C32	1.495 (7)
C14—O1	1.362 (3)	C31—H31A	0.9700
C14—C15	1.378 (4)	C31—H31B	0.9700
C15—C16	1.391 (3)	C32—C33	1.449 (9)
C15—H15	0.9300	C32—H32A	0.9700
C16—H16	0.9300	C32—H32B	0.9700
C17—O1	1.419 (4)	C33—C34	1.361 (9)
C17—H17A	0.9600	C33—H33A	0.9700
C17—H17B	0.9600	C33—H33B	0.9700
C17—H17C	0.9600	C34—O3	1.366 (7)
C21—C22	1.382 (4)	C34—H34A	0.9700
C21—C26	1.391 (4)	C34—H34B	0.9700
C21—P1	1.807 (2)	N1—N1^i^	1.417 (4)
C22—C23	1.396 (4)	N1—P1	1.678 (2)
C22—H22	0.9300	P1—Au1	2.2238 (11)
C23—C24	1.380 (4)	Cl1—Au1	2.2905 (11)
C23—H23	0.9300	Au1—Au1^i^	3.1222 (17)
			
N1—C1—H1A	109.5	C21—C26—H26	119.6
N1—C1—H1B	109.5	O2—C27—H27A	109.5
H1A—C1—H1B	109.5	O2—C27—H27B	109.5
N1—C1—H1C	109.5	H27A—C27—H27B	109.5
H1A—C1—H1C	109.5	O2—C27—H27C	109.5
H1B—C1—H1C	109.5	H27A—C27—H27C	109.5
C16—C11—C12	118.8 (2)	H27B—C27—H27C	109.5
C16—C11—P1	119.45 (19)	O3—C31—C32	105.7 (5)
C12—C11—P1	121.61 (19)	O3—C31—H31A	110.6
C13—C12—C11	120.4 (3)	C32—C31—H31A	110.6
C13—C12—H12	119.8	O3—C31—H31B	110.6
C11—C12—H12	119.8	C32—C31—H31B	110.6
C12—C13—C14	120.1 (3)	H31A—C31—H31B	108.7
C12—C13—H13	120.0	C33—C32—C31	105.2 (5)
C14—C13—H13	120.0	C33—C32—H32A	110.7
O1—C14—C15	124.4 (3)	C31—C32—H32A	110.7
O1—C14—C13	115.2 (3)	C33—C32—H32B	110.7
C15—C14—C13	120.4 (2)	C31—C32—H32B	110.7
C14—C15—C16	119.1 (3)	H32A—C32—H32B	108.8
C14—C15—H15	120.4	C34—C33—C32	104.1 (6)
C16—C15—H15	120.4	C34—C33—H33A	110.9
C11—C16—C15	121.2 (2)	C32—C33—H33A	110.9
C11—C16—H16	119.4	C34—C33—H33B	110.9
C15—C16—H16	119.4	C32—C33—H33B	110.9
O1—C17—H17A	109.5	H33A—C33—H33B	108.9
O1—C17—H17B	109.5	C33—C34—O3	113.0 (6)
H17A—C17—H17B	109.5	C33—C34—H34A	109.0
O1—C17—H17C	109.5	O3—C34—H34A	109.0
H17A—C17—H17C	109.5	C33—C34—H34B	109.0
H17B—C17—H17C	109.5	O3—C34—H34B	109.0
C22—C21—C26	118.8 (2)	H34A—C34—H34B	107.8
C22—C21—P1	119.4 (2)	N1^i^—N1—C1	116.05 (18)
C26—C21—P1	121.73 (19)	N1^i^—N1—P1	115.65 (17)
C21—C22—C23	121.0 (3)	C1—N1—P1	126.33 (17)
C21—C22—H22	119.5	C14—O1—C17	117.6 (3)
C23—C22—H22	119.5	C24—O2—C27	117.0 (3)
C24—C23—C22	119.2 (3)	C34—O3—C31	105.4 (5)
C24—C23—H23	120.4	N1—P1—C11	102.26 (11)
C22—C23—H23	120.4	N1—P1—C21	109.71 (12)
O2—C24—C23	124.8 (3)	C11—P1—C21	104.57 (11)
O2—C24—C25	114.8 (3)	N1—P1—Au1	110.83 (8)
C23—C24—C25	120.4 (2)	C11—P1—Au1	116.63 (9)
C26—C25—C24	119.6 (3)	C21—P1—Au1	112.20 (9)
C26—C25—H25	120.2	P1—Au1—Cl1	175.68 (3)
C24—C25—H25	120.2	P1—Au1—Au1^i^	88.241 (19)
C25—C26—C21	120.9 (2)	Cl1—Au1—Au1^i^	94.74 (2)
C25—C26—H26	119.5		
			
C16—C11—C12—C13	−1.9 (4)	C13—C14—O1—C17	170.4 (3)
P1—C11—C12—C13	174.3 (2)	C23—C24—O2—C27	−5.4 (5)
C11—C12—C13—C14	1.0 (5)	C25—C24—O2—C27	174.8 (3)
C12—C13—C14—O1	−178.9 (3)	C33—C34—O3—C31	26.4 (12)
C12—C13—C14—C15	0.6 (5)	C32—C31—O3—C34	−23.6 (9)
O1—C14—C15—C16	178.3 (3)	N1^i^—N1—P1—C11	−165.00 (15)
C13—C14—C15—C16	−1.3 (5)	C1—N1—P1—C11	31.7 (3)
C12—C11—C16—C15	1.3 (4)	N1^i^—N1—P1—C21	84.44 (16)
P1—C11—C16—C15	−175.0 (2)	C1—N1—P1—C21	−78.9 (3)
C14—C15—C16—C11	0.3 (4)	N1^i^—N1—P1—Au1	−40.01 (16)
C26—C21—C22—C23	1.4 (4)	C1—N1—P1—Au1	156.7 (2)
P1—C21—C22—C23	−175.7 (2)	C16—C11—P1—N1	76.7 (2)
C21—C22—C23—C24	−1.3 (4)	C12—C11—P1—N1	−99.5 (2)
C22—C23—C24—O2	−179.7 (3)	C16—C11—P1—C21	−168.9 (2)
C22—C23—C24—C25	0.1 (4)	C12—C11—P1—C21	14.9 (3)
O2—C24—C25—C26	−179.3 (2)	C16—C11—P1—Au1	−44.4 (2)
C23—C24—C25—C26	0.9 (4)	C12—C11—P1—Au1	139.4 (2)
C24—C25—C26—C21	−0.7 (4)	C22—C21—P1—N1	−162.70 (19)
C22—C21—C26—C25	−0.5 (4)	C26—C21—P1—N1	20.2 (2)
P1—C21—C26—C25	176.63 (19)	C22—C21—P1—C11	88.3 (2)
O3—C31—C32—C33	14.1 (8)	C26—C21—P1—C11	−88.8 (2)
C31—C32—C33—C34	1.1 (10)	C22—C21—P1—Au1	−39.0 (2)
C32—C33—C34—O3	−16.7 (13)	C26—C21—P1—Au1	143.88 (19)
C15—C14—O1—C17	−9.2 (5)		

Symmetry codes: (i) −*x*+1, *y*, −*z*+1/2.

## References

[bb1] Bruker (1998). *SMART-NT* Bruker AXS Inc., Madison, Wisconsin, USA.

[bb2] Bruker (1999). *SAINT-Plus* and *SADABS* Bruker AXS Inc., Madison, Wisconsin, USA.

[bb3] Farrugia, L. J. (1997). *J. Appl. Cryst.* **30**, 565.

[bb4] Farrugia, L. J. (1999). *J. Appl. Cryst.* **32**, 837–838.

[bb5] Holleman, A. F. & Wiberg, E. (2001). *Inorganic Chemistry*, p. 1248. San Diego: Academic Press.

[bb6] Kriel, F. H., Fernandes, M. A. & Caddy, J. (2010*a*). *Acta Cryst.* E**66**, o1270. 10.1107/S1600536810015886 PMC2979499 21579371

[bb7] Kriel, F. H., Fernandes, M. A. & Coates, J. (2010*b*). *Acta Cryst.* E**66**, m710. 10.1107/S1600536810019094 PMC2979462 21579343

[bb8] Kriel, F. H., Fernandes, M. A. & Coates, J. (2011*a*). *Acta Cryst.* E**67**, m42. 10.1107/S1600536810050506 PMC3050310 21522563

[bb9] Kriel, F. H., Fernandes, M. A. & Coates, J. (2011*b*). *Acta Cryst.* E**67**, m155. 10.1107/S1600536811000109 PMC3051471 21522837

[bb10] Macrae, C. F., Bruno, I. J., Chisholm, J. A., Edgington, P. R., McCabe, P., Pidcock, E., Rodriguez-Monge, L., Taylor, R., van de Streek, J. & Wood, P. A. (2008). *J. Appl. Cryst.* **41**, 466–470.

[bb11] Reddy, V. S., Katti, K. V. & Barnes, C. L. (1994). *Chem. Ber.* **127**, 1355–1357.

[bb12] Reddy, V. S., Katti, K. V. & Barnes, C. L. (1995). *Inorg. Chem.* **34**, 5483–5488.

[bb13] Sheldrick, G. M. (2008). *Acta Cryst.* A**64**, 112–122. 10.1107/S0108767307043930 18156677

[bb14] Slawin, A. M. Z., Wainwright, M. & Woollins, J. D. (2002). *J. Chem. Soc. Dalton Trans.* pp. 513–519.

